# *NTRK* rearrangements in a subset of NF1-related malignant peripheral nerve sheath tumors as novel actionable target

**DOI:** 10.1007/s00401-022-02515-3

**Published:** 2022-11-04

**Authors:** L. S. Hiemcke-Jiwa, M. T. Meister, E. Martin, M. P. Dierselhuis, L. M. Haveman, R. W. J. Meijers, B. B. J. Tops, P. Wesseling, P. J. van Diest, J. M. van Gorp, J. Y. Hehir-Kwa, I. A. E. M. van Belzen, J. J. Bonenkamp, M. M. van Noesel, U. Flucke, L. A. Kester

**Affiliations:** 1 Princess Máxima Center for Pediatric Oncology, Heidelberglaan 25, 3584 CS Utrecht, The Netherlands; 2 Department of Pathology, Utrecht University Hospital, Utrecht, The Netherlands; 3 Oncode Institute, Heidelberglaan 25, 3584 CS Utrecht, The Netherlands; 4 Department of Reconstructive Surgery, Utrecht University Hospital, Utrecht, The Netherlands; 5 Department of Pathology, Amsterdam University Medical Centers/Location VUmc, Amsterdam, The Netherlands; 6 Department of Pathology, St. Antonius Hospital, Nieuwegein, The Netherlands; 7 Department of Surgery, Radboud University Medical Center, Nijmegen, The Netherlands; 8 Division Imaging and Cancer, Utrecht University Hospital, Utrecht, the Netherlands; 9 Department of Pathology, Radboud University Medical Center, Nijmegen, The Netherlands

Malignant peripheral nerve sheath tumor (MPNST), often arising from a (plexiform) neurofibroma, is one of the hallmark complications of neurofibromatosis type 1 (NF1) characterized by aggressive behavior [10]. The genetic background is complex and heterogeneous, with the initiating biallelic *NF1* inactivation followed by a cascade of acquired mutations driving malignant progression. Amplification of receptor tyrosine kinase genes, have also been observed, and models demonstrated responses to the corresponding therapeutic blockades [7–9]. Fusion genes are rarely investigated in NF1-related MPNSTs [5]. We describe subclonal *NTRK* fusion genes in a subset of such tumors (Fig. 1), thereby potentially providing additional treatment options.

**Fig. 1:**
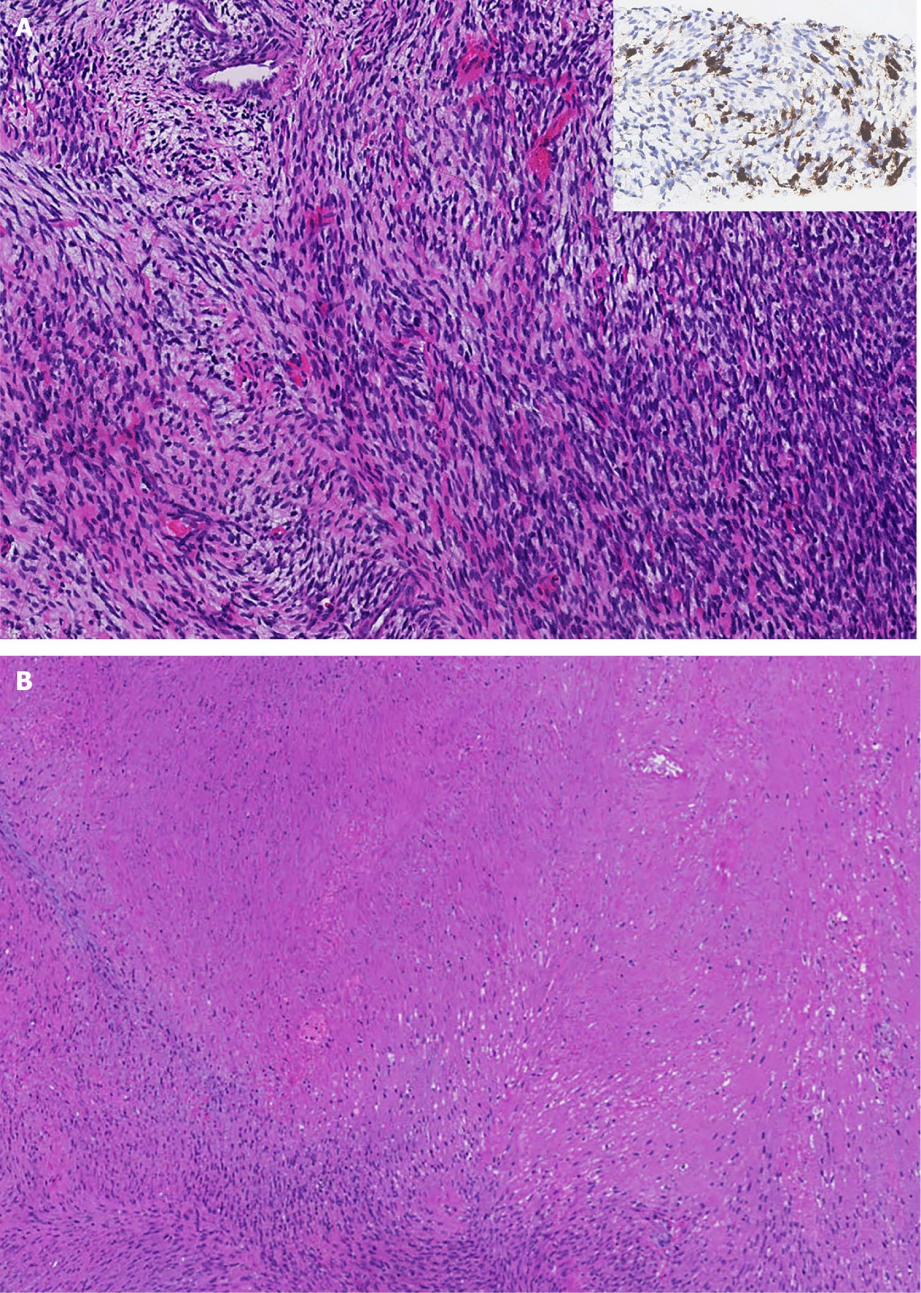
Morphological features of Case 1. **a** Primary biopsy showing an atypical cellular spindle cell tumor consistent with MPNST. Inset: partial pan-TRK expression. Magnification × 8. **b** First resection specimen depicted ~ 60% tumor necrosis. Magnification × 3

Three out of 21 (14%) cases of our cohort harbored a *NTRK1* fusion gene. The partner genes were *TPM3, LMNA* and *CACYBP* (Fig. 2). *TPM3::NTRK1* and *LMNA::NTRK1* are common driver fusion genes in *NTRK*-related spindle cell neoplasms [1], whereas *CACYBP::NTRK1* has not been reported in the literature so far. One could argue that these three tumors represent classical *NTRK*-rearranged spindle cell neoplasms unrelated to the NF1. Nonetheless, two tumors originated in a plexiform neurofibroma and harbored biallelic *NF1* mutations. The third case showed clinical signs of NF1, but failed to show two hits, possibly due to technical limitations (Tables 1, 2).

**Fig. 2:**
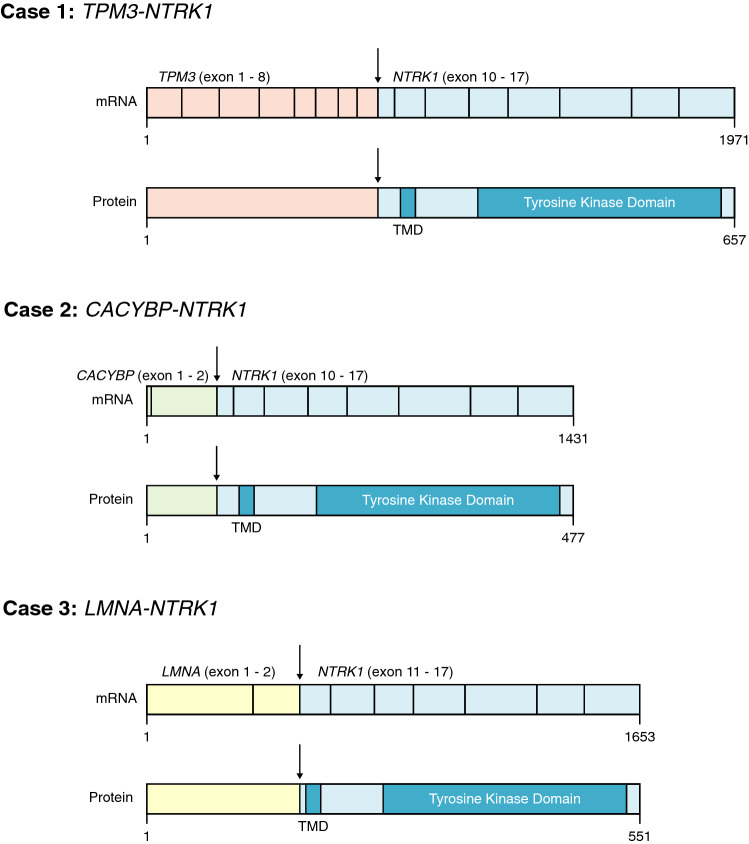
Detected fusion transcripts and the resulting fusion proteins in the three NF1-related MPNSTs with a *NTRK1* rearrangement. Functional regions and domains are annotated. Transmembrane Domain (TMD)

**Table 1:** Clinical characteristics of NF1 patients with MPNSTs harboring *NTRK* rearrangements

Case	Age	Location	Primary diagnosis	Metastases	Neo-adjuvant Therapy	Follow-up
1	16	Knee	MPNST ex plexiform neurofibroma	Yes (lung)	Trk-i	Aw/oD (18 months)
2	29	Sciatic nerve	MPNST ex plexiform neurofibroma	No	Radiotherapy	Aw/oD (8 years)
3	34	Quadriceps muscle	MPNST without signs of preexisting neurofibroma	No	None	Aw/oD (29 years)

*Aw/oD* alive without disease, *Trk-i* Trk-inhibitor

**Table 2:** Molecular characteristics of MPNSTs with *NTRK1* rearrangements

Case	Technique for fusion transcript analysis	Fusion gene	Other molecular alterations
1 (first biopsy)	RNA-seq	*TPM3::NTRK1* *exon 7 – exon 10*	Focal deletion 17p; second somatic mutation *NF1* c.7062_7063ins43 p.(Ser2355Valfs*7); 75% (WES)
2	RNA-seq	*CACYBP::NTRK1* *exon 2 – exon 10*	homozygous loss of *NF1* (CNV)
3	Archer	*LMNA::NTRK1* *exon 2 – exon 11*	Loss of one *NF1* allele; other allele not interpretable (CNV)

By WGS, FISH and/or immunohistochemistry, the *NTRK1* rearrangement presented as a subclonal molecular event in all three cases, further influencing MAPK signaling due to autoactivation of the corresponding transmembrane tyrosine kinase. *NTRK* genes, encoding for the neurotrophin family of growth factor receptors, have a crucial role in cell survival and proliferation, especially of neural tissue. Hence, it is not surprising that alterations in these genes can result in tumor development of MPNSTs [2].

Detection of *NTRK* chimeric fusion transcripts in NF1-associated MPNSTs might be of clinical importance as they may allow for targeted treatment with Trk-i as shown in one of our cases (Fig. 1a). While neurofibromin acts downstream of Trk, the sole blockade of the latter might be insufficient to fully abrogate MAPK signaling. In fact, a recent study showed that combined targeting of Trk and MEK, further downstream in the MAPK signaling pathway, in tumors harboring a *NTRK* fusion gene in combination with another activating alteration in the MAPK signaling pathway (i.e., activating *KRAS* and *BRAF* mutations) is paramount to prevent progression under Trk-i therapy and increase efficacy [3]. Whereas single agent treatment efficacy of MEK-i in NF1-related MPNSTs is questionable [4], the combination of a Trk-i and a MEK-i warrants further investigation.

In accordance with the intrinsic resistance against monotherapeutic Trk-i, the tumor of our treated case, initially showing good response, progressed during continuation of Trk-i treatment. A typical “escape” mutation in the kinase domain could not be detected by WES [2, 6]. Although the underlying resistance mechanism remains unclear so far, one could hypothesize that, besides another undetected mutation, quiescent cancer stem cells with specific genetic alterations are responsible for sustaining tumor growth [9].

Our study for the first time describes NF1-related MPNSTs harboring subclonal *NTRK* rearrangements with primarily good response to Trk-i treatment which could be an (additional) therapeutic agent.

## Supplementary Information

Below is the link to the electronic supplementary material.

Supplementary file1 (DOCX 40 kb)
