# Supplementary material for: NTRK rearrangements in a subset of NF1-related malignant peripheral nerve sheath tumors as novel actionable target
Source: Acta Neuropathol. 2022 Nov 4;145(1):149–52. doi: 10.1007/s00401-022-02515-3 (PMC9807516; doi:10.1007/s00401-022-02515-3)
Supplement: Supplementary file 1 — Supplementary file1 (DOCX 40 kb) [file 401_2022_2515_MOESM1_ESM.docx]

**Acta Neuropathologica**

***NTRK* rearrangements in a subset of NF1-related malignant peripheral nerve sheath tumors as novel actionable target**

L. S. Hiemcke-Jiwa^*1,5^, M. T. Meister^2,3*^, E. Martin^4^, M. P. Dierselhuis^1^, L. M. Haveman^1^, R. W. J. Meijers^5^, B. B. J. Tops^1^, P. Wesseling^1,9^, P. J. van Diest^5^, J. M. van Gorp^6^, J. Y. Hehir-Kwa^2^, I. A. E. M. van Belzen^2^, J. J. Bonenkamp^7^, M. M. van Noesel^1,8^, U. Flucke^$1,9^, L. A. Kester^$1^

Address of correspondence:

Laura S. Hiemcke-Jiwa

Princess Máxima Center for Pediatric Oncology, Heidelberglaan 25, 3584 CS, Utrecht, The Netherlands

**Materials and Methods**

*Sample selection and case characteristics*

31 formalin-fixed paraffin embedded (FFPE) MPNST samples from 25 NF1-patients were collected from our files. 19 samples from 16 patients yielded RNA of sufficient quality for molecular analyses, although in only 3 samples assay quality was of sufficient quality to exclude false negative results.

*Immunohistochemistry (IHC)*

Slides were stained using an automated Ventana tissue stainer (BenchMark Ultra, Roche). The following antibodies were used: S100 (ready to use, 4C4.9, Roche), SOX10 (ready to use EP268, Roche), CD34 (ready to use, QBend/10, Roche), pan-Trk (Abcam, clone EPR17341, 1:500) and H3K27me3 (Cell Signaling, clone C36B11, 1:25).

*Fluorescence in situ hybridization (FISH) analysis*

Dual-color FISH analysis was performed on FFPE tissue using a break-apart probe for *NTRK1* (ZytoLight Z-2167-50) according to the manufacturer’s protocol. At least 100 nuclei of neoplastic cells per sample were counted.

*Targeted mRNA sequencing*

RNA was isolated from FFPE with the Maxwell RSC 48 Instrument (Promega, Madison, Wisconsin, USA) using the RNA FFPE kit (promega) according to standard protocol. cDNA libraries were generated with 250 ng RNA using the Archer FusionPlex Lung Kit for Ion Torrent (Archer, Boulder, CO, USA) containing primers for *NTRK1* (exon 2, 4-6, 8, 10-13), *NTRK2* (exons 5, 7, 9,1 1-17) and *NTRK3* (exons 4, 7, 10, 12-16). Sequencing was performed with the Ion S5 instrument (Thermo Fisher Scientific, Waltham, Massachusetts, USA) and Archer analysis software (version 6.0) was used for data analysis.

*mRNA sequencing (RNA-seq)* was performed as previously described [5, 6, 10].

*Whole exome sequencing (WES)* was done according to standard procedures

[1, 8, 10].

*Whole genome sequencing (WGS)*

WGS was performed on the libraries generated for WES prior to the WES capture. Sequencing and primary data analysis of the WGS data were done as for the WES data.

*Structural variant analysis from WGS data*

Structural variants (SVs) were inferred from paired tumor-normal WGS using Manta (version 1.6) [4], DELLY (version 0.8.1) [9] and GRIDSS (version 2.7.2) [3]. Structural variants supporting the creation of fusion transcripts were identified using Fusion-sq (https://www.biorxiv.org/content/10.1101/2021.08.31.458342v1).

*Copy number variation (CNV) analysis* was performed as previously described[7].

*Data availability*

The process to make the sequencing data publicly available at the European Genome-Phenome Archive (EGA) has been started and will be completed prior to the publication.

**Results**

*Clinicopathological and molecular characteristics of the three patients with MPNSTs harboring a NTRK rearrangement*

**Case 1** was a 16-year-old male NF1 patient with a second MPNST of the knee, diagnosed on biopsy (Figure 1a) [2].

RNA-seq depicted a *TPM3::NTRK1* fusion transcript (exon 7 - exon 10) with 1.97 FFPM (fusion fragments per million). The *NTRK1* rearrangement was also identified by WGS revealing a 2.7Mb inversion (chr1: 154,166,247 – 156,874,006) with an allele frequency of 5-8% depending on the structural variation algorithm, suggesting that it is a subclonal event. A germline deletion and an additional somatic mutation in *NF1* (c.7062_7063ins43 p.(Ser2355Valfs*7)) (allele frequency 75%; Table 2) were found as well. FISH analysis confirmed the *NTRK1* break (18% of tumor cells). Immunohistochemistry showed partially positive pan-Trk (Figure 1a inset).

A Trk-i was administered leading to reduction of tumor size and surgery with positive margins was subsequently performed.Trk-i was continuated but the tumor progressed and a second resection was done with negative margins. Several weeks thereafter a newly developed lung metastasis was completely resected. The patient is currently in complete remission (follow-up 18 months; Table 1).

Histologically, the first resection specimen had signs of regression (fibrosis and necrosis) of at least 60% without immunohistochemical detection of pan-Trk positive cells (Figure 1b).

In the second resection specimen mainly vital tumor was observed, morphologically and immunohistochemically similar to the first biopsy, including foci with pan-Trk positive tumor cells.

RNA-seq on the first resection specimen revealed no *NTRK1* fusion transcripts.

In the second resection specimen *NTRK1* rearrangement was detected using FISH (14% of tumor cells), corresponding with the areas with positive pan-Trk immunohistochemical staining.

**Case 2** was a 29-year-old man known with NF1 and a MPNST originating from a plexiform neurofibroma attached to the sciatic nerve. The tumor was marginally resected and adjuvant radiotherapy was administered. Follow-up was uneventful over a period of 8 years.

Morphologically, a preexisting neurofibroma was apparent with transition into a cellular neoplasm consistent with MPNST.

RNA-seq yielded a *CACYBP::NTRK1* fusion (exon 2 – exon 10). FISH analysis confirmed *NTRK1* rearrangement matching with the areas of positive pan-Trk immunohistochemistry. CNV analysis demonstrated a homozygous deletion of *NF1*.

**Case 3** was a 34-year-old male with multiple cutaneous neurofibromas in his thigh and a MPNST in the quadriceps muscle. The tumor was completely resected without signs of recurrence or metastases after 29 years of follow-up.

Histomorphology and immunohistochemistry confirmed the diagnosis [2]. Also, partial immunohistochemical positivity for pan-Trk was seen.

Targeted RNA-seq, performed on FFPE material, revealed a *LMNA::NTRK1* fusion (exon 2 -exon 11). *NTRK1* FISH was not interpretable. CNV analysis showed loss of one *NF1* allele; deletion of the other allele was not detectable, possibly due to undetectable small deletions and mutations.

**References**

1. Benjamin D, Sato T, Cibulskis K, Getz G, Stewart C, Lichtenstein LT. Calling Somatic SNVs and Indels with Mutect2. bioRxiv (2019).

2. Board WCoTE. Soft Tissue and Bone Tumours: International Agency for Research on Cancer; 2020.

3. Cameron DL, Schröder J, Penington JS, Do H, Molania R, Dobrovic A, et al. GRIDSS: sensitive and specific genomic rearrangement detection using positional de Bruijn graph assembly. Genome Res (2017);27:2050-2060.

4. Chen X, Schulz-Trieglaff O, Shaw R, Barnes B, Schlesinger F, Källberg M, et al. Manta: rapid detection of structural variants and indels for germline and cancer sequencing applications. Bioinformatics (2016);32:1220-1222.

5. Dobin A, Davis CA, Schlesinger F, Drenkow J, Zaleski C, Jha S, et al. STAR: ultrafast universal RNA-seq aligner. Bioinformatics (2013);29:15-21.

6. Hehir-Kwa JY, Koudijs MJ, Verwiel ETP, Kester LA, van Tuil M, Strengman E, et al. Improved Gene Fusion Detection in Childhood Cancer Diagnostics Using RNA Sequencing. JCO Precis Oncol (2022);6:e2000504.

7. Koelsche C, Schrimpf D, Stichel D, Sill M, Sahm F, Reuss DE, et al. Sarcoma classification by DNA methylation profiling. Nat Commun (2021);12:498.

8. McLaren W, Gil L, Hunt SE, Riat HS, Ritchie GR, Thormann A, et al. The Ensembl Variant Effect Predictor. Genome Biol (2016);17:122.

9. Rausch T, Zichner T, Schlattl A, Stütz AM, Benes V, Korbel JO. DELLY: structural variant discovery by integrated paired-end and split-read analysis. Bioinformatics (2012);28:i333-i339.

10. Wingett SW, Andrews S. FastQ Screen: A tool for multi-genome mapping and quality control. F1000Res (2018);7:1338.
